# Remarks on the History and Nature of the Dolor Faciei

**Published:** 1801-09

**Authors:** 


### *Remarks on the History and Nature of the Dolor Faciei.*

( Concluded from p. 158—164 of our laft. )

3. *Exanthematous Acrimony.*

EXANTHEMATA have been obferved in this difeafe either in the face itfelf or in fome other part of the body. Mr. ANDRE obferved miliary eruptions in the face, upon the difappearance of which the pain began to ceafe; but when they broke out again, the pain recurred with the former violence. BONNARD and LEGAVAN relate inftances of herpetic eruptions on the arm having preceded the diforder; and the German tranflator of Dr. FOTHERGILL's works faw it appear after a retrograde itch: The pain difcontinued when the itch broke out again, but as foon as it was removed by proper remedies, the pain immediately returned; and the difeafe lafted in this way from the nineteenth year of age to the twenty-feventh.

4. *Scrophulous acrimony.* MENOURET and SELLE, loc. cit. affign this as a caufe of the difeafe in two inftances.

5. *Cartarrhous acrimony.* VOGLER and PUJOL, loc. cit.

6. RHEUMATIC MATTER. It is not improbable that this is a moft frequent caufe of that painful affection, which we find likewife confirmed by the authority of experience and obfervation.

7. *Venereal poison.* M. SIEBOLD is not inclined to admit this as a caufe of the above difeafe, becaufe the venereal pain is always feverer in the night than in the day, without intermiffions, and feems to be more feated in the bones than in the muscular and nervous parts. We have, befides, no obfervation of the venereal poifon being affigned as the caufe of that pain, if we except Mr. WATON. (*Journal de Medicine*, 1793, *month of March.*) It has been, however, obferved, that the difeafe was fuccefsfully treated with mercurials; but this can by no means ferve as an argument for the venereal nature of the diforder.

8. *Preceding serous profluvia suppressed.* Dr. LENTIN relates a cafe in which a purulent falivation preceded the pain, by the appearance of which, the paroxyfm of the pain became lefs vehement and fhorter: In another cafe the pain fucceeded a running of the ears; and Dr. Thilenius faw a patient who had a kind of gonorrhœa previoufly to the pain, which began to run again as foon as the pain had ceafed.

VAN SWIETEN (*Commentar. in Aphorism. Boerhaave*, §. 757) and DAVIDSON (*loc. citat.*) have obferved a pain in the face fimilar to an ague, which, however, effentially differs, according to Dr. FOTHERGILL, from the diforder we are fpeaking of. The patient of VAN SWIETEN had a very fevere hemicrania, lafting every day for eight hours, which, after this time, abated by degrees. At the firft attack the pain appeared in the place where the branch of the fifth pair iffues from the foramen fupraorbitale: In another cafe a topical febrile pulfe was remarked at the affected place: The difeafe was cured by the bark. The typic character of this pain, and the termination of its paroxyfm, with a ftrong perfpiration, fufficiently diftinguifhes it from the true Dolor Faciei.

CASE I. A country woman, near forty, of a thin habit, had been feized with a very violent and unufual pain affecting the *canthus* of the mouth, when fhe was ftooping to cut grafs. Having been afflicted with it for nearly a month, fhe was fo much relieved by topical venæfection, and leeches applied behind the ears, that fhe could attend to her ufual bufinefs till the next fpring, but the pain never wholly went off. About this time, when fhe felt herfelf big with child, the pain returned with greater violence than ever; fhe had feveral teeth drawn out, but to no purpofe. However, when a few months of her pregnancy were elapfed, the pain ceafed entirely, and fhe was luckily delivered of a healthy boy. Four months after the lying-in, the pain attacked her for the third time with the utmoft violence, and continued in this way till fhe came under the care of Mr. SIEBOLD. Her ftrength and health were much impaired, and fhe had been treated during the whole time with gummi guaiacum, aconitum, and linimentum volatile, but without the leaft fuccefs. The feat of the pain was not far from the right angle of the mouth, and confined to fo fmall a fpot, that it could be covered with the top of a finger, though fometimes it reached down to the *maxilla inferior*. Notwithftanding this, the fpot was not at all changed, but perfectly fimilar to the other fide; the preffure with a finger caufed no pain, and only the glands of the cheek were a little fwollen. At the edge of the under lip, not far from the affected place, fhe had a fmall pea-like foft tumour, of a blueifh colour, in which, however, fhe felt not the leaft pain; fmall ulcerations were, befides, perceived at the inner fide of the lips, moft probably occafioned by the teeth biting the place during the paroxyfm: the molar teeth were, for the moft part, wanting. The pain never returned at a certain time, and lafted fometimes only a few minutes; but at another time, day and night, with equal violence, fo that the patient could not fleep for nearly three months. Speaking or chewing would immediately excite the pain. The mouth became dry during the paroxyfm, and likewife the nofe, and the eyes began to fhed tears as foon as as the vehemence of the paroxyſm was over: The patient endeavoured to mitigate the violence of the pain by preſſing the tongue againſt the affected place, by rubbing it with linen, and by contortions of the mouth and face. The taſte remained unaltered, except that ſhe ſeemed now to like ſweet things better than ſour and ſalt victuals, of which ſhe had been very fond before: She became more coſtive when the diſeaſe got worſe. Her abdomen was ſometimes ſwelled, but without any pain: the urine of a pale colour. In her younger years ſhe had been much ſubject to tooth-ach and ſwellings of the cheeks. The plan adopted for her cure was the following, viz. milk diet with ſoft eggs, and the following medicine, which ſhe commenced on the 11th of October, viz. ℞. *Extract cicutæ*, drachm ij. *Pulv. herb. cicutæ* ʒß. *M. F. pill. pond.* gr. iij. S. four times a day, four pills. ℞. Decoct. cort. Peruviani unc. viij. gum arabic, dr. iij. ſyrup de althæa unc. Ms. to take every three hours half a tea cup full. As the pains, however, increaſed, and the pulſe became full and tenſe, ſhe diſcontinued the bark.

12th. The night very reſtleſs and painful; her pulſe is full and hard; ſkin ſoft and moiſt; no ſtool ſince three days, on which account ſhe took one drachm and a half of ſal polychreſtum.

13th. A hard ſtool; continued the pills, and a decoction of hemlock was ordered to be kept in the mouth for ſome time.

14th. Another doſe of the ſalt for the coſtiveneſs, and every two hours three pills.

15th. A tolerable night; a little ſleep; gentle evacuation of pituitous matter after adminiſtering an enema.

16th. The pain continued, on which account an emplaſtr. de cicut. was applied.

19th. The pain had conſiderably diminiſhed, and even ceaſed for three hours; coſtiveneſs; increaſed the doſe of the pills to one more; conſiderable abatement of the pain towards the evening.

20th. She had ſlept very quietly till midnight.

21ſt. Some pain when ſhe began to ſpeak; the ulcerations in the mouth, abovementioned, healed, ſkin moiſt; pulſe frequent, without being tenſe or hard; five pills at a time. She continued in this way the *cicuta* and the milk diet, till ſhe was attacked, *November* 14, with a febrile paroxyſm, accompanied with head-ache, vertigo, congeſtions of blood towards the head, and great anxiety: She perſpired at the ſame time ſo much, that ſhe was obliged to change her linen ſeveral times in the night. Theſe ſymptoms, however, were removed within four days, by bliſters and proper evacuants; but inſtead of them an an abfcefs arofe at the upper arm, attended with a copious fuppuration; after which fhe felt fo much relief, that fhe omitted rubbing the affected place; was able to fpeak flowly, and to chew without exciting the pain.

*December* 13. From this date to the 21ft of January fhe took twice, daily, one fcruple of the powder of valerian, which did her much good, the pains abating more and more of their violence, fo that it was now thought proper to leave the reft to Nature. On January the 25th, however, fome fevere paroxyfms of the pain returning, fhe was ordered to rub in a mercurial ointment twice a day, which being continued for fome days, the pain began to ceafe, and fhe felt nothing of it for two days, which continued fo till fhe was difmiffed, about the middle of February. The abfcefs had healed, but it was thought proper to make an iffue near the place. Menftruation had not appeared after the laft pregnancy.

Case II.—The patient was a woman fifty years of age, of a robuft habit, whofe menftruation had ceafed twelve years ago. As a cook, fhe had been much expofed to the heat of the fire; her face was rather tender, but the fkin relaxed. The pain firft attacked her when fhe was wafhing, and rubbing her face with a linen handkerchief; it came fo fudden, and with fuch violence, that fhe fcreamed. It continued thus, day and night, for a whole year, but it never prevented her from fleep: The pain extended itfelf over the whole right fide, running down the temples and cheeks, and fhe felt likewife a fingular fenfation on the forehead of that fide, (*afflatio, fufurrus.*) She could open neither the lips nor the jaws, without exciting the moft violent pains; but a confiderable preffure on the gums, or the external parts, did not produce the pain. She had fuffered much tooth-ach from her younger years, on which account fhe had all the teeth of the upper jaw drawn out. Several years previous to her being feized with the tooth-ach, fhe was attacked with fevere pains in the joints of the fingers, which fhe fuppofed to have ceafed by venæfection. Four years ago a particular eruption arofe on the right arm, fimilar to miliaria, and a year after fhe had been feized with fevere pains in the joint of the foot; the eruption appeared again a fhort time before the *Dolor Faciei* came on. Thefe painful affections in the joints, and the miliary eruptions, feem to prove an affinity of this Dolor Faciei with the gout, particularly as we know, by the obfervations of Burserius and Strack, that topical miliary exanthemeta are frequently owing to arthritic matter.

Case III. A gentleman, of a full habit and ftrong make, much fubject to colds, had the misfortune of running his forehead againft againſt a tree, which immediately excited the *Dolor Faciei*, that laſted ſixteen years, extending to the noſe and down the cheeks. The pain was deſcribed by the patient as ſtinging, ſometimes as pinching: The affected ſide was ſomewhat decayed and emaciated. Though the patient had never had tooth-ach, yet he had ſome teeth drawn out, but without any effect. Moving the lips would immediately excite the pain; and, likewiſe, the leaſt draught of air frequently brought it on. A preſſure on the nervus infraorbitalis, at its exitus, would prevent the pain from extending itſelf over the face. After ſome time, during which he uſed the *Portland* powder, he experienced a conſiderable ſenſeleſsneſs in his hands, and ſometimes an inſupportable burning in the ſoles of the feet.

This caſe will be more fully deſcribed in future by Prof. *Iſenflamm*, of Erlangen, who has communicated a ſketch of it to our author.

